# Multiplex plasma protein profiling identifies novel markers to discriminate patients with adenocarcinoma of the lung

**DOI:** 10.1186/s12885-019-5943-3

**Published:** 2019-07-29

**Authors:** Dijana Djureinovic, Victor Pontén, Per Landelius, Sahar Al Sayegh, Kai Kappert, Masood Kamali-Moghaddam, Patrick Micke, Elisabeth Ståhle

**Affiliations:** 1 Department of Immunology, Genetics and Pathology, Uppsala University, 751 85 Uppsala, Sweden; 2 Department of Surgical Sciences, Uppsala University, Uppsala, Sweden; 3 Institute of Laboratory Medicine, Clinical Chemistry and Pathobiochemistry, Charité - Universitätsmedizin Berlin, corporate member of Freie Universität Berlin, Humboldt-Universität zu Berlin, and Berlin Institute of Health, Berlin, Germany; 4 Department of Immunology, Genetics and Pathology, Science for Life Laboratory, Uppsala University, Uppsala, Sweden

**Keywords:** Lung cancer, Tumor markers, Blood, Plasma, Screening, Biomarker

## Abstract

**Background:**

The overall prognosis of non-small cell lung cancer (NSCLC) is poor, and currently only patients with localized disease are potentially curable. Therefore, preferably non-invasively determined biomarkers that detect NSCLC patients at early stages of the disease are of high clinical relevance. The aim of this study was to identify and validate novel protein markers in plasma using the highly sensitive DNA-assisted multiplex proximity extension assay (PEA) to discriminate NSCLC from other lung diseases.

**Methods:**

Plasma samples were collected from a total of 343 patients who underwent surgical resection for different lung diseases, including 144 patients with lung adenocarcinoma (LAC), 68 patients with non-malignant lung disease, 83 patients with lung metastasis of colorectal cancers and 48 patients with typical carcinoid. One microliter of plasma was analyzed using PEA, allowing detection and quantification of 92 established cancer related proteins. The concentrations of the plasma proteins were compared between disease groups.

**Results:**

The comparison between LAC and benign samples revealed significantly different plasma levels for four proteins; CXCL17, CEACAM5, VEGFR2 and ERBB3 (adjusted *p*-value < 0.05). A multi-parameter classifier was developed to discriminate between samples from LAC patients and from patients with non-malignant lung conditions. With a bootstrap aggregated decision tree algorithm (TreeBagger), a sensitivity of 93% and specificity of 64% was achieved to detect LAC in this risk population.

**Conclusions:**

By applying the highly sensitive PEA, reliable protein profiles could be determined in microliter amounts of plasma. We further identified proteins that demonstrated different plasma concentration in defined disease groups and developed a signature that holds potential to be included in a screening assay for early lung cancer detection.

**Electronic supplementary material:**

The online version of this article (10.1186/s12885-019-5943-3) contains supplementary material, which is available to authorized users.

## Background

Lung cancer remains the leading cause of cancer-related deaths worldwide. The prognosis is poor across all stages with five-year survival rates of 13% [1]. In advanced disease, where systemic therapy is the only option, the patient’s five-year survival rate is as low as 4% [1]. To detect lung cancer at earlier stages, screening with low-dose computed tomography (LDCT) is recommended for high-risk individuals with a history of extensive smoking and with an age between 55 and 80 years [2]. LDCT was shown to reduce lung cancer mortality by 20% [3]. Beside the high costs, the high false positive rate particularly limits the value of this method, and a benefit for a broader use beyond high risk patients has not been proven [4, 5]. Thus, other inexpensive and non-invasive approaches are called for to improve the usefulness of lung cancer screening programs in individuals without clinical symptoms with the aim to be more accurate. Blood-based assays seem to be the most promising options for screening purposes and to improve early and true cancer detection rates in symptomatic and non-symptomatic individuals, since they are easily accessible, fast and relatively inexpensive. One of the most established blood-based biomarkers is prostate specific antigen (PSA), although its use is controversial due to low specificity and potential over-diagnosis with consecutive invasive therapies and costs [6, 7]. Besides screening purposes, plasma or serum biomarkers are primarily applied for disease surveillance and to monitor response to therapy. An example is carcinoma antigen 125 (CA125), which is upregulated in particular in gynecological cancer types and its abundance in serum is used to detect relapse, and to monitor response to treatment in patients with ovarian cancer [8]. Importantly, increasing levels of CA125 indicate recurrence three to four months before it is clinically evident or detectable by imaging (lead time) [9]. Further, specificity and sensitivity might be enhanced by multi-parameter approaches.

Many studies have aimed to develop and validate clinical tests for early diagnosis of lung cancer, including blood-based assays to detect microRNAs, cell-free circulating tumor DNA, autoantibodies or proteins with increased levels in the plasma or serum of cancer patients compared to those of healthy individuals [10–14]. Indeed, some of these tests are demonstrated to provide additional information to computer tomography (CT) screening, but none is sufficiently validated to be used alone in the clinical routine [15, 16].

The aim of this study was to assess plasma derived from patients with lung adenocarcinoma (LAC), colorectal metastasis (CRC met), typical carcinoids, and a control group with non-malignant lung diseases using the novel multiplex proximity extension assay (PEA). Further, we tested which of the 92 oncology-related proteins is best suitable alone or in combination to discriminate patients with lung cancer from patients with other thoracic malignancies and, in particular, from patients with benign lung disease. This should ultimately lead to the identification of plasma biomarkers for early detection of lung cancer.

## Methods

### Patient samples

Blood samples were collected from patients admitted to Uppsala University Hospital, Sweden, Department of Thoracic Surgery undergoing surgical resection for therapeutic or diagnostic purpose of different lung diseases between 2002 and 2013. Patients´ characteristics of the 343 samples consisting of 144 patients with LAC, 68 patients with benign lung diseases, 83 patients with CRC met and 48 patients with typical primary carcinoids originating from the lung included in this study are listed in Table 1. The diagnosis of the 68 patients with benign lung diseases is described in Table 2.

**Table 1:** Clinical characteristics of patient’s samples included in the study

	LAC (%)	Benign (%)	CRC met (%)	Typical carcinoid (%)
Number. of patients	144 (100)	68 (100)	83 (100)	48 (100)
Gender				
Female	81 (56)	34 (50)	41 (49)	31 (65)
Male	63 (44)	34 (50)	42 (51)	17 (35)
Age				
≤ 70	80 (56)	52 (76)	59 (71)	43 (90)
> 70	53 (37)	16 (24)	24 (29)	5 (10)
Smoking status				
Smoker	130 (90)	41 (60)	43 (52)	22 (46)
Non-smoker	11 (8)	27 (40)	36 (43)	23 (48)
NA	3 (2)	0 (0)	4 (5)	3 (6)
Stage				
IA	56 (39)	–	–	–
IB	18 (12)	–	–	–
IIA	17 (12)	–	–	–
IIB	14 (10)	–	–	–
IIIA	33 (23)	–	–	–
IIIB	3 (2)	–	–	–
IV	2 (1)	–	–	–
NA	1 (1)	–	–	–

*LAC* Lung adenocarcinoma, *CRC met* Colorectal metastasis

**Table 2:** The diagnosis of benign samples

Diagnosis	Patients
Benign tumors	25
Inflammatory disease^a^	5
Necrotizing granulomatous inflammation	6
Other	6
Pneumonia / acute inflammation	12
Pneumonia / chronic inflammation	14

^a^Inflammatory disease with potential systemic background

### Plasma analysis

EDTA-Plasma levels of 92 proteins were analyzed using Olink Multiplex Oncology II panel (Additional file 1: Table S1) based on the PEA technology as previously described [17, 18]. Briefly, in PEA one microliter plasma is incubated with a set of probes, each consisting of an antibody conjugated to a specific DNA oligonucleotide. Once a protein is recognized by a pair of probes, the DNA oligonucleotides of the antibody pairs, now in proximity, are allowed to hybridize to each other and are extended by enzymatic polymerization. The newly formed DNA molecule is then amplified and quantified by real-time PCR. The PCR results were analyzed as normalized protein expression (NPX) values on a log2 scale. NPX values were obtained by normalizing Cq-values against extension controls, inter-plate control and a correction factor. A high NPX value corresponds to a high protein concentration and expresses relative quantification between samples but represents no absolute quantification. Details about data validation, limit of detection (LOD), specificity and reproducibility can be obtained via Olink’s homepage (http://www.olink.com). Of the 92 proteins five proteins were not detected in at least one of the samples (S100A4, CTSV, MICA/B, CEACAM5 and MUC16). Thus, 95% of all proteins were detected in all samples and 93% of samples had values above LOD for all 92 proteins.

### Development of discriminative classifier - statistical analysis

Comparative analysis between the patients’ groups was carried out using Wilcoxon test with the R statistical software version 3.2.5. Multiple testing corrections were done with the Benjamini-Hochberg method, and an adjusted *p*-value < 0.05 was considered significant. Hierarchical clustering analysis, the development of the classifiers and receiver operating characteristic (ROC) curves were performed using Matlab R2017b. A signature was developed to discriminate between LAC and non-malignant samples as well as carcinoids and metastases. The classification learner from the statistics and machine learning toolbox was applied to 80% of the data randomly selected and validated on the remaining 20%. We compared the performance of the TreeBagger class [19], K-Nearest Neighbour (KNN) [20], support vector machine (SVM) [21] and linear discriminant analysis (LDA) [22]. The method used for further analysis was the TreeBagger function that is an aggregated bootstrapping function using the random forest algorithm. To optimize performance and minimize the out of bag classification error, 5000 trees were initially created and weighted. Two thousand five hundred trees were then used for further analysis. An algorithm was then used to extract the classifier’s three best discriminating proteins with associated protein cut-off values from each tree giving the size of the best predicted group.

The pseudo code is provided as additional material (Additional file 2, Pseudocode).

## Results

### Comparison of plasma protein levels in different patient groups

An analysis of the 92 proteins (Additional file 1: Table S1) was performed in plasma samples from 144 patients with LAC, 68 patients with benign lung disease, 83 patients with CRC met and 48 typical carcinoids, and the protein levels in LAC were compared to those in the other patient groups (Table 1).

**Fig. 1:**
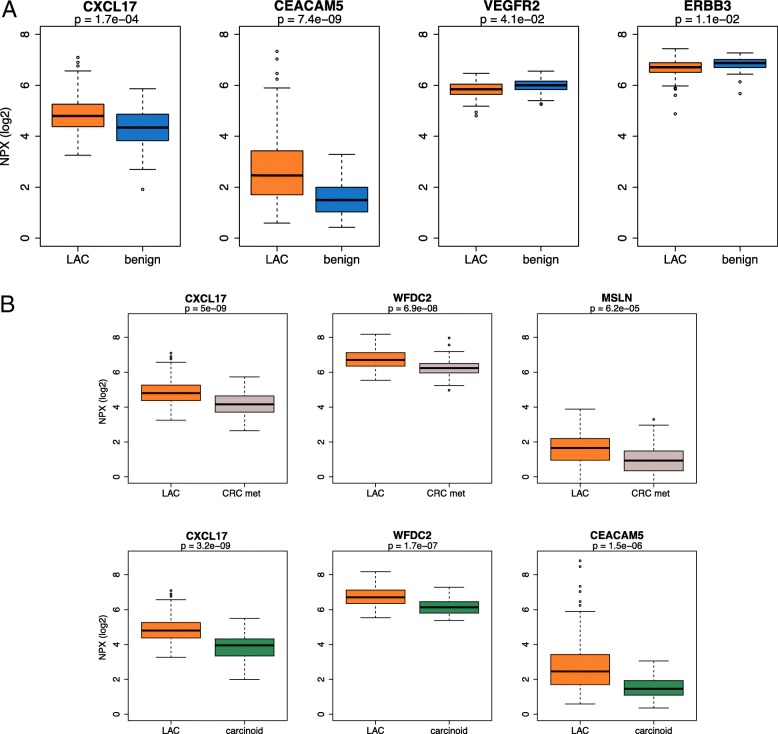
Plasma protein level differences**. a** Boxplots illustrating the levels of the four proteins with largest difference between lung adenocarcinoma (LAC) patients and patients with benign lung diseases. **b** Boxplots illustrating the levels of the three proteins with lowest adjusted *p*-value from the comparison of lung adenocarcinoma (LAC) with colorectal metastasis (CRC met) and typical carcinoids, respectively. *P*-values are adjusted for multiple testing. NPX: normalized protein expression values

**Fig. 2:**
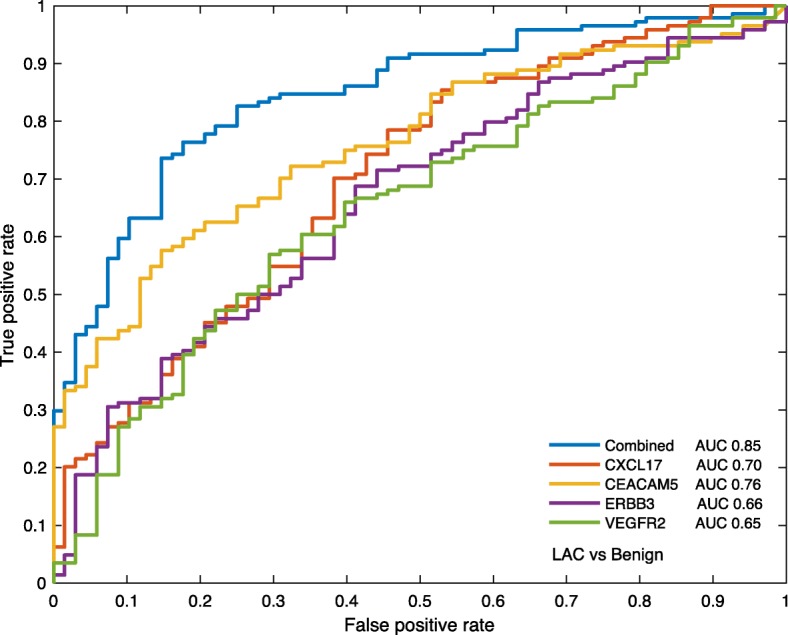
Receiver operating characteristic (ROC) curve for lung adenocarcinoma (LAC) and benign samples**.** The ROC curve was based on 20% LAC samples and 20% of benign samples visualizing the discriminatory model obtained with single proteins CXCL17, CEACAM5, ERBB3, VEGFR2 and the combination of all four proteins

When the levels of the 92 proteins were compared between LAC and benign lung diseases, the concentration of 30 proteins (33%) were found to be different for the two groups. After rigorous adjustment for multiple testing, the levels of four proteins remained significantly different. The plasma levels of c-x-c motif chemokine ligand 17 (CXCL17) and carcinoembryonic antigen related cell adhesion molecule 5 (CEACAM5) were significantly higher in LAC compared to non-malignant controls (adjusted *p*-value < 0.001, for both comparisons), while the levels of vascular endothelial growth factor receptor 2 (VEGFR2) and erb-b2 receptor tyrosine kinase 3 (ERBB3) were lower in LAC samples compared to those in non-malignant controls (adjusted *p*-value = 0.04 and 0.01, respectively). Non-malignant controls were either of inflammatory (e.g. pneumonia and abscess; Table 2) or non-inflammatory conditions (e.g hamartoma and benign solitary fibrous tumor). When inflammatory conditions were excluded from this comparison, only two proteins (CXCL17 and CEACAM5) demonstrated different plasma levels between LAC and non-malignant controls (adjusted *p*-value < 0.01 for both proteins) (Additional file 1: Table S1). A comparison between CRC met and LAC samples revealed different plasma levels for five proteins (WFDC2, MSLN, CXCL17, CEACAM5 and VEGFR2; adjusted *p*-value < 0.01 for all five proteins). Different levels of 12 proteins were observed when the plasma of patients with typical carcinoids was compared to plasma of LAC patients (adjusted *p*-value < 0.05, for all comparisons; (Additional file 1: Table S1). Despite that the plasma levels of several proteins differed significantly between all groups, the overlap of individual protein values was too high to distinguish between cancer and benign samples on a single protein level as indicated by boxplots in Fig. 1, and confusion matrix in Table 3. ROC curve of the four proteins (CXCL17, CEACAM5, VEGFR2 and ERBB3) revealed that CEACAM5 has the highest area under the curve (AUC) value for the single markers, and that the combination of all four proteins gives a slightly better classification of LAC and controls. However, even with best cut-point selection, the performance was relatively low to predict cancer (Fig. 2)

**Table 3:** Comparison of single protein classifiers with median as cut-off^a^

CEACAM5					
Specificity	1.00		Low	High	
Sensitivity	0.26	Benign	68	0	True
PPV	1.00	LAC	113	31	outcome
NPV	0.38		Predicted outcome		
Accuracy	0.47				
CXCL17					
Specificity	0.66		Low	High	
Sensitivity	0.60	Benign	45	23	True
PPV	0.79	LAC	58	86	outcome
NPV	0.44		Predicted outcome		
Accuracy	0.62				
VEGFR2			Low	High	
Specificity	0.35	Benign	24	44	True
Sensitivity	0.40	LAC	87	57	outcome
PPV	0.56		Predicted outcome		
NPV	0.22				
Accuracy	0.38				
ERBB3			Low	High	
Specificity	0.40	Benign	36	54	True
Sensitivity	0.44	LAC	81	63	outcome
PPV	0.54		Predicted outcome		
NPV	0.31				
Accuracy	0.42				

^a^The median protein level was used as cut-off (high vs low) to determine group affiliation

*PPV* Positive predictive value, *NPV* Negative predictive value, *LAC* Lung adenocarcinoma

### Development of LAC specific protein signature

**Fig. 3:**
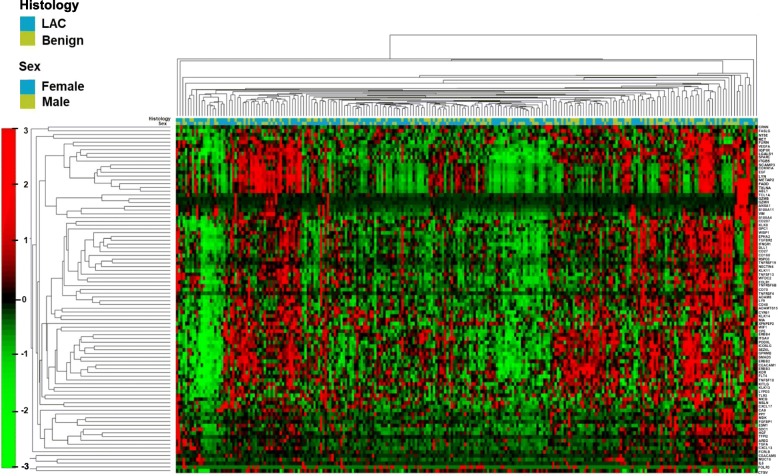
Hierarchical cluster analysis based on plasma protein levels**.** Hierachical cluster analysis of 144 lung adenocarcinoma (LAC) and 68 patients with benign lung disease based on all 92 analyzed proteins

**Fig. 4:**
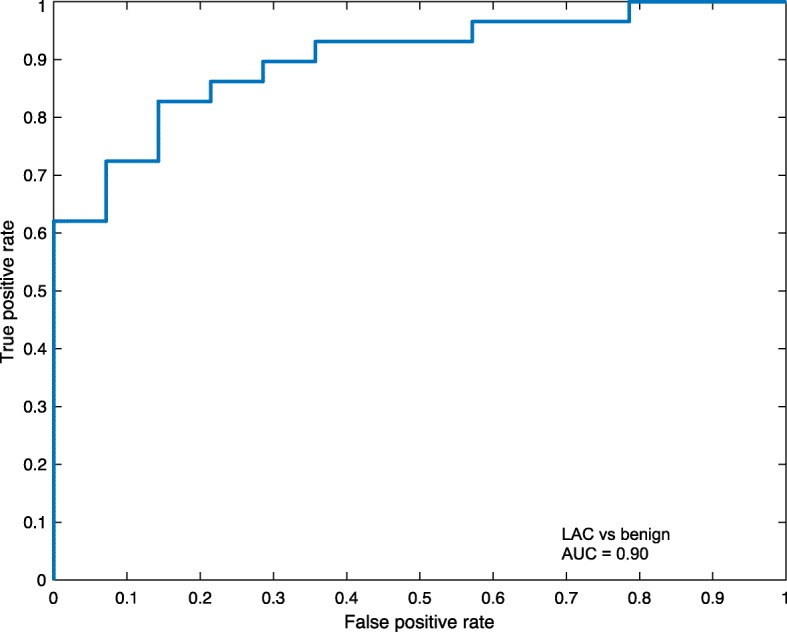
Receiver operating characteristic (ROC) curve for lung adenocarcinoma (LAC) and benign samples**.** The ROC curve was based on 20% of LAC samples and 20% of benign samples visualizing the discriminatory model obtained with TreeBagger resulting in an area under the curve (AUC) of 0.90

To evaluate whether the combination of proteins can discriminate benign from cancer cases, we performed a hierarchical cluster analysis based on all 92 plasma proteins (Fig. 3). Although several clusters with general higher (red) or lower (green) expression could be distinguished, this unbiased approach did not separate between LAC and benign lung diseases, when the plasma profile of all protein levels were analyzed. Therefore, we used the data set to develop a discriminating model with 4 different classification learners (TreeBagger, KNN, SVM and LDA). In this comparison, the TreeBagger classifier showed the overall best performance. When the TreeBagger model was applied on the remaining 20% of samples (29 cancer and 14 controls) a specificity of 64% and a sensitivity of 93% was obtained (Table 4). A ROC curve based on the TreeBagger model of the remaining 20% of LAC samples and 20% of the benign samples resulted in an AUC of 0.90 (Fig. 4).

**Table 4:** Comparison of performance of different classification models^a^

	Specificity	Sensitivity	PPV	NPV	Accuracy
TreeBagger class	0.64	0.93	0.84	0.82	0.84
K-Nearest Neighbour	0.71	0.62	0.82	0.48	0.65
Support vector machine	0.00	1.00	0.67	Div/0	0.67
Linear discriminant analysis	0.71	0.59	0.81	0.45	0.63

^a^For training 80% of lung adenocarcinomas (LAC) and benign were used and 20% of both groups were used for validation. *PPV* Positive predictive value, *NPV* Negative predictive value

In depth analysis revealed three proteins with the highest discriminating power: CEACAM5, WFDC2 and TCL1A. Applied on our patient cohort, 45% of LAC patients, 18% of the patients with CRC met, but none of the patients with benign lung diseases showed increased levels of these three proteins (Table 5).

**Table 5:** Performance of 3 protein classifier. The cut-off was chosen (CEACAM5 > 4.92, WFDC2 > 75.57, TCL1A > 8.34) to best separate benign and cancer cases and LAC and CRC met cases

Signature vs Benign					
Specificity	1.00	Benign	68	0	True
Sensitivity	0.45	Tumor	79	65	outcome
PPV	1.00		Predicted outcome		
NPV	0.46				
Accuracy	0.63				
Signature vs CRC met					
Specificity	0.82	CRC met	68	15	True
Sensitivity	0.45	LAC	79	65	Outcome
PPV	0.81		Predicted outcome		
NPV	0.46				
Accuracy	0.59				

*PPV* Positive predictive value, *NPV* Negative predictive value, *LAC* Lung adenocarcinoma, *CRC met* colorectal metastasis

A classifier was separately developed including 80% of the LAC with stage I (*n* = 59) and the benign samples (*n* = 54). When the TreeBagger model was applied on the validation set of the remaining 20% of samples (15 cancer and 14 controls) a specificity of 93% and a sensitivity of 86% was obtained (Table 6). The proteins with most discriminatory power in this analysis were FCRLB, VEGFR-3 and TXLNA that were not detectable (below the medium value as cut-off) in 39% of the LAC samples and 2% of the benign samples.

**Table 6:** TreeBagger model to separate stage I lung adenocarcinoma from the benign on 29 samples for validation

Signature vs. Benign					
Specificity	0.93	Benign	13	1	Predicted
Sensitivity	0.87	LAC	2	13	outcome
PPV	0.93		True outcome		
NPV	0.87				
Accuracy	0.90				

Benign: samples with both inflammatory and non-inflammatory conditions: *LAC* Stage 1, *PPV* Positive predictive value, *NPV* Negative predictive value

Furthermore, in a separated analysis the LAC with stage I (*n* = 59) was compared with a subgroup of benign samples not associated with inflammation (*n* = 22). When the TreeBagger model applied on the remaining 20% of samples (15 cancer and 6 controls) a specificity of 67% and a sensitivity of 93% was obtained (Table 7). The proteins with most discriminatory power in this analysis were CYR61, WFDC2 and SCAMP3 that were detected at high levels (above medium value as cut-off) in 46% of the LAC samples and in none of the benign samples.

**Table 7:** TreeBagger model to separate stage I lung adenocarcinoma from those with non-inflammatory benign lung disease on 21 samples for validation

Signature vs. Benign					
Specificity	0.67	Benign	4	2	Predicted
Sensitivity	0.93	Tumor	1	14	outcome
PPV	0.88		True outcome		
NPV	0.80				
Accuracy	0.86				

Benign: the samples with non-inflammatory conditions: *LAC* Stage 1, *PPV* Positive predictive value, *NPV* Negative predictive value

**Fig. 5:**
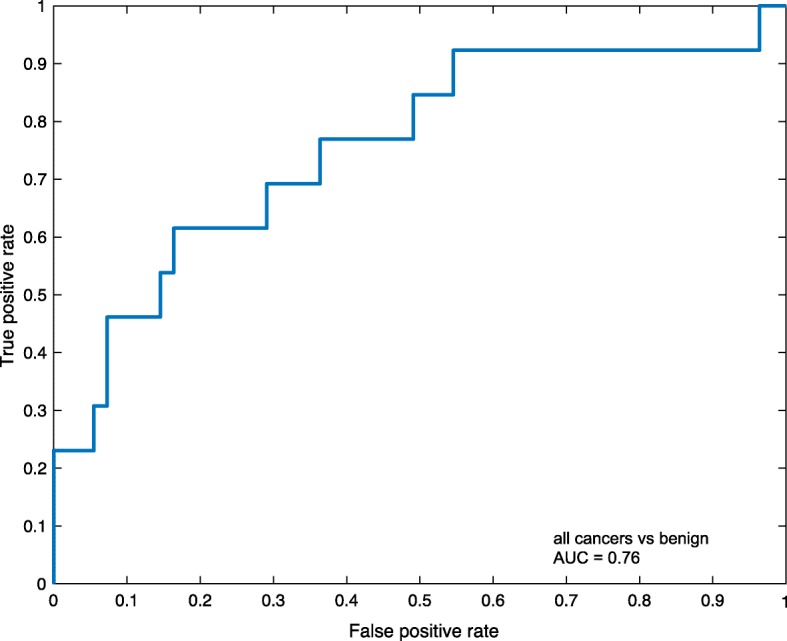
Receiver operating characteristic (ROC) curve for all cancer samples and benign samples. The ROC curve was based on 20% of all cancer samples (lung adenocarcinoma, colorectal metastases and typical carcinoids) and 20% of benign samples visualizing the discriminatory model obtained with TreeBagger resulting in an area under the curve (AUC) of 0.76

Finally, we investigated whether a classifier could separate benign disease from the combined group of LAC, CRC met and typical carcinoids. Using the TreeBagger model, we reached a high specificity of 98% but a low sensitivity of 14% between malignant and benign lung diseases (Table 8). A ROC curve based on the TreeBagger model of the remaining 20% of all cancer samples (LACs, CRC met and typical carcinoids) and 20% of the benign samples resulted in an AUC of 0.76 (Fig. 5).

**Table 8:** TreeBagger model to separate tumor from benign lung disea*se on 59 samples for validation*

Specificity	0.98				
Sensitivity	0.14	Tumors^a^	44	1	True
PPV	0.67	Benign	12	2	outcome
NPV	0.79		Predicted outcome		
Accuracy	0.78				

^a^All tumors: lung adenocarcinoma, colorectal metastasis, typical carcinoids; *PPV* Positive predictive value, *NPV* Negative predictive value

## Discussion

Assays based on blood samples hold great potential as primary screening methods for cancer, because they are non-invasive, relatively inexpensive and easily applicable in clinical practice. However, specific or sensitive blood-based tumor markers, such as PSA for prostate cancer or Septin 9 methylated DNA for colorectal cancer [23], has yet not been identified in lung cancer. The technology applied in this study is a multiplex assay analyzing 92 proteins simultaneously and is based on the PEA [17]. The PEA technology offers several advantages compared to conventional immunoassays: (1) The technique is ultrasensitive allowing detection of proteins in pico- to femtomolar concentrations. This is non-inferior or better than most commercially available single-plex immunoassays [17]. (2) The use of DNA-conjugated pairs of antibodies minimizes reported signals due to unspecific cross-reactivities, thus providing high specificity for each analyzed protein. (3) The required plasma volume is minimal with only one μl, avoiding extensive blood sampling and saving valuable blood samples in clinical studies and samples from biobanks. (4) The possibility of multiplexing without compromising specificity and sensitivity facilitates disease specific assays to identify signatures for different clinical needs. Therefore, the assay seems to be an advanced and beneficial tool to analyze protein profiles as potential cancer biomarkers. The assay is currently only used for screening and research purposes. However, the use as companion diagnostics in several clinical trials, mostly in the context of heart diseases, indicates its potential as a diagnostic tool in the clinical setting [24]. In addition, after identification of the most relevant proteins, a dedicated panel with a few proteins or a single protein assay might be established.

The selected targeted proteins are generally involved in tumor immunity, chemotaxis, vascular and tissue remodeling, apoptosis and tumor metabolism. With this background, it is important to consider that the protein panel was not developed explicitly for lung cancer.

Despite this more general assay set-up, altogether 30 out of 92 proteins (33%) demonstrated differential plasma concentration between lung cancer samples and samples derived from patients with non-malignant lung disease. Even after rigorous adjustment for multiple testing, four proteins remained significantly different (CEACAM5, CXCL17, VEGFR2 and ERBB3).

CEACAM5 (often only abbreviated as CEA) is expressed in normal epithelial cells and overexpressed in the majority of carcinomas including lung carcinomas. CEACAM5 has been reported to play a role in innate and adaptive immunity in non-malignant lung epithelia [25]. It functions as an intracellular adhesion molecule in tumors and may directly promote tumor development and drive metastasis [26]. CEACAM5 is a clinically well-established tumor antigen [27–29] and is demonstrated to have a great concentration variation between cancer and controls. In lung cancer, the clinical value of CEACAM5 is limited because of its insufficient sensitivity and specificity, but it is often used in combination with other tumor markers [30]. Importantly, CEACAM5 was also significantly increased when LAC samples were compared to plasma from patients with CRC met, indicating its specificity for LAC.

CXCL17 belongs to the family of chemokines that are chemoattractants for monocytes, macrophages and dendritic cells. In non-malignant tissues, the expression of CXCL17 is predominantly found in mucosal linings including lung airways and is considered to have an anti-microbial function [31]. Elevated CXCL17 expression has been observed in patients with both non-malignant [31] and malignant diseases, where it is thought to directly promote tumor progression. For lung cancer, the tumor promoting effect has so far only been observed in vitro but the elevated plasma levels in lung cancer patients, even compared to pure inflammatory diseases in our study, supports the concept that CXCL17 is not only an inflammatory mediator but may be directly involved in tumorigenesis [32, 33].

In contrast to CEACAM5 and CXCL17, two markers demonstrated lower levels in the plasma of cancer patients: VEGFR2 acts as a cell-surface receptor for vascular endothelial growth factors (VEGFA, VEGFC and VEGFD), and is involved in angiogenesis in both physiological and pathological conditions. Serum levels of VEGFR2 have previously been evaluated in lung cancer with conflicting results; one study has reported higher [34] and one study - in agreement with our study - demonstrated lower levels [35] in NSCLC compared to controls. In both of these studies the control samples were from healthy individuals. Lower mRNA expression levels have been observed in lung cancer samples compared to non-malignant tissue using RNA-sequencing [36]. ERBB3 (alias HER3), a member of the epidermal growth factor receptor family, is expressed in normal bronchial epithelia and has been shown to be overexpressed in several cancers including lung cancer [37]. ERBB3 is considered to play a role in proliferation, differentiation and other normal processes and is associated with cancer cell growth including lung cancer [38].

While upregulation of CEACAM5 and CXCL17 seems to have a biological explanation, the underlying mechanism of the lower systemic levels of both important cancer related receptor tyrosine kinases, VEGFR2 and ERBB3, in lung cancer patients, remains elusive. However, we believe that these four proteins as well as several others from the top of the protein list represent promising candidates for further evaluation as tumor markers in plasma and/or tissue.

Although none of the proteins was sufficient as single tumor marker to distinguish lung cancer patients from non-malignant diseases, the combination of markers is the most obvious strategy to increase the performance of a screening assay. Today neural network-based models represent the state of the art in the analysis of multidimensional data sets [39]. In our study, the TreeBagger decision tree was used and could discriminate between LAC and benign diseases with a sensitivity of 93% and a specificity of 64%, with a relatively high negative predictive value of 82%. This is of particular importance, because individuals with malignancies should not accidently be missed by a negative result. When we performed an analysis on only LAC with stage I versus benign, the performance of the classifier was similar. The three proteins with the highest discriminatory power, however, differed. This may be due to that the pattern of protein levels bears the discriminatory power and not the single protein. In comparison to other studies, evaluating blood-based cancer assays, our results seem promising. A previous study analyzed classical tumor markers in a large set of 530 lung cancer patients and 229 healthy controls. By combining CEA, NSE, CYFRA21-1, CA125, CA199 and ferritin in different combinations, a sensitivity of up to 94% was reached, but with a low specificity between 26 and 45% [30]. The study of Bigbee also used a multiplex strategy, including 70 cancer-related tumor markers quantified with a bead-based immunoassay. They identified 10 tumor markers that were combined to a classifier [13]. This classification resulted in 73% sensitivity and 93% specificity in a validation data set of 30 lung cancer and 30 control samples. None of the ten proteins were included in our panel. More recently, several mass spectrometry (MS) methods have been applied for screening purposes. In a notable systematic approach, the group of Kearny [40] developed a 13-protein classifier and reached between 71 and 100% sensitivity with a specificity of 28–56%. Another small MS-study reported a sensitivity of 95% and specificity of 85% [41]. An earlier study applying the surface-enhanced laser desorption/ionization (SELDI) technology on serum samples yielded a sensitivity of 87% and specificity of 80% [42]. In an metabolomic strategy Maeda et al., evaluated amino acid profiles in the plasma of lung cancer patients and controls with a promising accuracy [43]. However, these MS-based techniques are costly, time-consuming and thus difficult to implement in routine clinical diagnostic, and accreditation is more complex than with relatively simple immunoassays [44, 45]. In this light, we believe the assay used in our study has a realistic potential to be further developed to a routine clinical screening assay. It is likely that its performance can be considerably improved, when the most significant proteins from our study would be complemented by defined promising tumor markers from other studies.

Although the results reported herein support the usefulness of the PEA for screening purpose, our findings should be regarded as descriptive. We only included lung adeno carcinomas and did not analyze the complete set of NSCLCs, which would have been the preferred strategy. Also, a complete independent patient cohort, confirming the findings of the original data set is necessary. Nevertheless, we applied adequate statistical analyses, including stringent adjustment for multiple testing and statistical modelling of training and validation cohort. Another point of concern is that this is a retrospective study and the evaluation of a diagnostic assay should ideally be done in a prospective fashion. Another study limitation might be that our control group is not optimally balanced. The group consists of consecutive patients that underwent surgery for different medical reasons, only some of them with primary suspicion for cancer, that were diagnosed with a non-malignant disease after operation. These controls did not perfectly represent individuals that would be considered as candidates for lung cancer screening (current or former heavy smokers between 50 and 70 years of age [3]). Therefore, an optimization and an extension of the control group seems warranted for a further validation of the assay. Since inflammation is a part of the malignant process, we included samples from patients with inflammatory conditions as controls and not samples from healthy donors because we aimed to identify proteins that also can discriminate cancer from the inflammatory process. A complete representation of NSCLC, and not only lung adenocarcinomas, and extended group of controls would naturally be the subject for a future validation study.

Today, LDCT screening is the only recommended method for lung cancer screening to reduce mortality in a high-risk population. A blood-based test may be applied before the CT-screening, decreasing unnecessary CT scans, or after the CT scan avoiding unnecessary intervention in benign diseases. Both strategies require accurate test methods that ultimately have to be validated for its diagnostic use in prospective clinical trials.

## Conclusion

Our study evaluated the diagnostic performance of a multiplex plasma protein immunoassay in a clinically well-characterized cohort of NSCLC patients. We identified several proteins that showed different plasma concentrations between patients with LAC and other lung diseases and developed a classifier that could identify lung cancer in a risk population. The results indicate that this technique in combination with an optimal protein panel has the potential to serve as a screening assay for early detection of lung cancer.

## Additional files

Additional file 1: **Table S1.** Proteins included in the Olink Multiplex Oncology II panel and the corresponding *p*-value when comparing protein levels in LAC vs. benign, CRC metastases and typical carcinoids. (PDF 223 kb)

Additional file 2: **Pseudo code:** Pseudo code for the TreeBagger algorithm, which was used to develop a multi-parameter classificator. (DOCX 14 kb)

## Data Availability

The data analyzed are available from the corresponding author on reasonable request. The datasets supporting the conclusions of this article are included within the article and its Additional files.

## Abbreviations

CA125: Carcinoma antigen 125
CEACAM5: Carcinoembryonic antigen related cell adhesion molecule 5
CRC met: Colorectal metastasis
CXCL17: c-x-c motif chemokine ligand 17
ERBB3: erb-b2 receptor tyrosine kinase 3
KNN: k-nearest neighbor
LAC: Lung adenocarcinoma
LDA: Linear discriminant analysis
LDCT: Low-dose computed tomography
LOD: Limit of detection
MS: Mass spectrometry
NPX: Normalized protein expression
PEA: Proximity extension assay
PSA: Prostate specific antigen
ROC: Receiver operating characteristic
SVM: Support vector machine
VEGFR2: Vascular endothelial growth factor receptor 2
