# Supplementary material for: Multiplex plasma protein profiling identifies novel markers to discriminate patients with adenocarcinoma of the lung
Source: BMC Cancer. 2019 Jul 29;19:741. doi: 10.1186/s12885-019-5943-3 (PMC6664554; doi:10.1186/s12885-019-5943-3)
Supplement: Supplementary file 1 — Table S1. Proteins included in the Olink Multiplex Oncology II panel and the corresponding p-value when comparing protein levels in LAC vs. benign, CRC metastases and typical carcinoids. (PDF 223 kb) [file 12885_2019_5943_MOESM1_ESM.pdf]

Additional file 1: Table S1. Proteins included in the Olink Multiplex Oncology II panel and the corresponding p-value when comparing protein levels in LAC vs benign, CRC metastases and typical carcinoids.

Protein name	Gene name	mean NPX LAC	SD LAC	mean NPX benign	SD benign	mean NPX benign*	SD benign*	mean NPX CRC met	SD CRC met	mean NPX typical carcinoid	SD typical carcinoid	p-val LAC vs benign	p-val LAC vs benign*	p-val LAC vs CRC met	p-val LAC vs typical carcinoid
Vascular endothelial growth factor receptor 2	VEGFR-2	5.84	0.30	5.99	0.27	5.91	0.26	5.66	0.33	5.86	0.33	0.00 ↓	0.24	0.00 ↑	0.72
Receptor tyrosine-protein kinase erbB-3	ERBB3	6.68	0.34	6.84	0.26	6.68	0.29	6.62	0.30	6.71	0.29	0.00 ↓	0.97	0.14	0.87
VEGF-co regulated chemokine 1	CXCL17	4.85	0.70	4.28	0.74	4.18	0.68	4.19	0.60	3.89	0.80	0.00 ↑	0.00 ↑	0.00 ↑	0.00 ↑
Carcinoembryonic antigen-related cell adhesion molecule 5	CEACAM5	2.81	1.55	1.56	0.66	1.48	0.66	2.02	1.36	1.56	0.68	0.00 ↑	0.00 ↑	0.00 ↑	0.00 ↑
Carcinoembryonic antigen-related cell adhesion molecule 1	CEACAM1	6.30	0.28	6.44	0.26	6.30	0.25	6.25	0.28	6.28	0.28	0.00	0.99	0.15	0.44
Vascular endothelial growth factor receptor 3	VEGFR-3	4.97	0.36	5.13	0.41	4.94	0.47	4.86	0.39	4.90	0.36	0.00	0.81	0.03	0.18
WAP four-disulfide core domain protein 2	WFDC2	6.73	0.52	6.51	0.52	6.43	0.50	6.26	0.52	6.15	0.48	0.00	0.00	0.00 ↑	0.00 ↑
Transmembrane glycoprotein NMB	GPNMB	5.09	0.30	5.21	0.26	5.10	0.21	5.15	0.27	5.10	0.23	0.01	0.84	0.12	0.80
Nectin-4	PVRL4	5.03	0.63	4.82	0.50	4.69	0.54	4.75	0.56	4.69	0.44	0.01	0.00	0.00	0.00 ↑
TNF-related apoptosis-inducing ligand	TNFSF10	6.55	0.40	6.70	0.34	6.57	0.35	6.60	0.40	6.62	0.37	0.01	0.81	0.75	0.44
Interleukin-6	IL-6	2.47	1.28	2.15	1.54	1.79	1.04	2.01	1.12	1.52	1.13	0.01	0.01	0.02	0.00 ↑
Midkine	MK	5.96	0.70	5.74	0.59	5.60	0.53	5.85	0.74	5.65	0.58	0.01	0.01	0.23	0.00
Tumor necrosis factor ligand superfamily member 13	TNFSF13	7.61	0.42	7.48	0.46	7.38	0.43	7.53	0.49	7.28	0.42	0.01	0.00	0.22	0.00 ↑
R-spondin-3	RSPO3	2.79	0.64	2.57	0.52	2.52	0.57	2.79	0.56	2.63	0.50	0.01	0.02	0.46	0.19
Disintegrin and metalloproteinase domain-containing protein 8	ADAM 8	3.34	0.41	3.49	0.41	3.28	0.34	3.24	0.39	3.31	0.34	0.01	0.45	0.11	0.60
Amphiregulin	AR	1.25	0.69	1.03	0.51	0.96	0.51	1.22	0.83	0.98	0.57	0.02	0.01	0.39	0.00
Fibroblast growth factor-binding protein 1	FGF-BP1	3.87	0.68	4.01	0.49	3.84	0.40	3.90	0.69	3.86	0.36	0.02	0.73	0.78	0.48
Kallikrein-14	hK14	5.45	0.58	5.23	0.57	5.20	0.54	5.41	0.57	5.53	0.44	0.02	0.05	0.87	0.50
Galectin-1	Gal-1	5.08	0.38	5.20	0.34	5.09	0.35	5.01	0.34	4.95	0.36	0.02	0.85	0.18	0.04
SPARC	SPARC	5.20	0.35	5.30	0.36	5.22	0.40	5.16	0.36	5.14	0.34	0.02	0.59	0.51	0.24
Tissue factor pathway inhibitor 2	TFPI-2	6.80	0.84	6.51	0.53	6.41	0.54	6.67	0.72	6.46	0.52	0.02	0.01	0.32	0.00
ICOS ligand	ICOSLG	2.61	0.36	2.74	0.31	2.61	0.27	2.62	0.28	2.65	0.25	0.02	0.75	0.97	0.64
Tumor necrosis factor receptor superfamily member 6B	TNFRSF6B	3.50	0.74	3.29	0.65	3.06	0.56	3.39	0.75	3.09	0.67	0.02	0.00	0.22	0.00 ↑
CD48 antigen	CD48	5.28	0.50	5.45	0.46	5.25	0.36	5.23	0.51	5.31	0.50	0.02	0.78	0.69	0.85
Integrin alpha-V	ITGAV	1.91	0.38	2.02	0.32	1.96	0.33	1.96	0.36	1.92	0.26	0.03	0.52	0.34	0.98
Proto-oncogene tyrosine-protein kinase receptor Ret	RET	2.36	0.55	2.53	0.63	2.25	0.50	2.52	0.45	2.69	0.47	0.03	0.41	0.02	0.00 ↓
T-lymphocyte surface antigen Ly-9	LY9	3.98	0.46	4.14	0.41	3.97	0.34	3.95	0.48	4.00	0.46	0.03	0.83	0.49	0.88
Stem cell factor	SCF	7.59	0.66	7.78	0.57	7.79	0.50	7.78	0.50	7.69	0.51	0.04	0.15	0.02	0.28
Tumor necrosis factor ligand superfamily member 6	FASLG	7.54	0.57	7.72	0.50	7.73	0.48	7.63	0.53	7.89	0.55	0.04	0.19	0.18	0.00 ↓
Protein CYR61	CYR61	4.48	0.51	4.36	0.42	4.24	0.47	4.50	0.56	4.37	0.52	0.04	0.01	0.84	0.19
Cathepsin L2	CTSV	2.23	0.55	2.31	0.45	2.40	0.41	2.24	0.47	2.51	0.43	0.06	0.04	0.72	0.00 ↓
Integrin beta-5	ITGB5	6.74	0.46	6.82	0.44	6.71	0.44	6.71	0.48	6.57	0.45	0.08	0.88	0.84	0.03
Kallikrein-13	KLK13	3.91	0.64	3.80	0.61	3.65	0.63	3.77	0.62	3.92	0.52	0.09	0.04	0.04	0.64
Tumor necrosis factor receptor superfamily member 19	TNFRSF19	3.06	0.62	2.96	0.53	2.88	0.52	3.01	0.54	2.86	0.51	0.09	0.04	0.56	0.02
Methionine aminopeptidase 2	MetAP2	4.46	0.91	4.66	0.86	4.74	0.82	4.39	0.86	4.45	0.80	0.09	0.11	0.68	0.94
Protein S100-A11	S100A11	2.30	0.71	2.11	0.49	2.19	0.68	2.10	0.45	2.17	0.78	0.10	0.17	0.11	0.06
Pancreatic prohormone	PPY	3.55	1.49	3.18	1.18	3.42	0.99	3.47	1.28	3.33	1.89	0.11	0.70	0.80	0.15
Seizure 6-like protein	SEZ6L	3.94	0.44	4.02	0.32	3.96	0.30	4.05	0.44	3.99	0.34	0.12	0.80	0.06	0.49
MHC class I polypeptide-related sequence A and B	MIC-A/B	2.78	1.63	3.00	1.39	2.75	1.31	2.83	1.58	3.09	1.25	0.14	0.58	0.71	0.74
Glypican-1	GPC1	2.84	0.41	2.92	0.44	2.71	0.47	2.90	0.39	2.89	0.38	0.14	0.17	0.09	0.62
Kallikrein-11	hK11	4.53	0.54	4.43	0.48	4.39	0.49	4.32	0.46	4.42	0.53	0.14	0.08	0.00	0.14
Podocalyxin	PODXL	2.53	0.29	2.61	0.27	2.55	0.25	2.54	0.26	2.55	0.22	0.14	0.98	0.96	0.90
Mothers against decapentaplegic homolog 5	MAD homolog 5	2.54	0.31	2.58	0.27	2.51	0.23	2.50	0.29	2.45	0.26	0.14	0.53	0.34	0.03
Receptor tyrosine-protein kinase erbB-4	ERBB4	3.69	0.41	3.76	0.31	3.70	0.32	3.67	0.40	3.71	0.29	0.15	1.00	0.88	0.90
Ly6/PLAUR domain-containing protein 3	LYPD3	2.79	0.43	2.87	0.51	2.72	0.45	2.69	0.45	2.94	0.42	0.17	0.63	0.18	0.12
Carbonic anhydrase 9	CA9	1.78	0.85	1.60	0.78	1.67	0.98	2.03	1.04	1.44	1.10	0.17	0.47	0.14	0.00
Syndecan-1	SYND1	5.84	0.77	5.71	0.75	5.42	0.63	5.70	0.72	5.64	0.61	0.18	0.00	0.25	0.27
Folate receptor alpha	FR-alpha	5.72	0.52	5.63	0.41	5.50	0.42	5.53	0.55	5.54	0.47	0.21	0.04	0.01	0.02
Interferon gamma receptor 1	IFN-gamma-R1	2.71	0.38	2.78	0.32	2.64	0.26	2.76	0.42	2.74	0.35	0.23	0.21	0.21	0.68
WNT1-inducible-signaling pathway protein 1	WISP-1	3.46	0.51	3.52	0.48	3.30	0.45	3.46	0.58	3.26	0.48	0.24	0.25	0.82	0.02
Insulin-like growth factor 1 receptor	IGF1R	2.40	0.35	2.45	0.33	2.33	0.27	2.33	0.37	2.33	0.30	0.24	0.45	0.18	0.16
Mesothelin	MSLN	1.62	0.92	1.48	0.84	1.31	0.76	1.01	0.75	0.98	0.75	0.26	0.12	0.00 ↑	0.00 ↑
Protein S100-A4	S100A4	1.45	0.60	1.54	0.53	1.52	0.60	1.31	0.44	1.42	0.53	0.26	0.59	0.05	0.29
Delta-like protein 1	DLL1	8.13	0.45	8.22	0.39	8.04	0.38	8.10	0.45	8.03	0.41	0.27	0.20	0.88	0.14
Pro-epidermal growth factor	EGF	7.30	1.67	7.05	1.74	7.03	1.77	7.17	1.84	7.28	1.61	0.30	0.45	0.77	0.89
Vascular endothelial growth factor A	VEGF-A	9.16	0.58	9.10	0.60	8.92	0.57	8.90	0.57	8.80	0.50	0.30	0.02	0.00	0.00 ↑

Secretory carrier-associated membrane protein 3	SCAMP3	3.53	1.42	3.71	1.54	3.56	1.43	3.35	1.42	3.32	1.28	0.34	1.00	0.44	0.42
5'-nucleotidase	5-NT	8.67	0.57	8.80	0.75	8.55	0.68	9.03	0.81	8.74	0.64	0.35	0.24	0.00	0.49
Hepatocyte growth factor	HGF	5.41	0.68	5.30	0.48	5.11	0.45	5.29	0.61	5.11	0.59	0.37	0.02	0.33	0.00
Granzyme H	GZMH	3.14	1.46	3.06	1.48	3.10	1.96	3.23	1.65	3.03	1.48	0.37	0.14	0.94	0.41
Wnt inhibitory factor 1	WIF-1	4.31	0.49	4.33	0.38	4.29	0.35	4.33	0.52	4.39	0.48	0.43	0.99	0.69	0.37
Ephrin type-A receptor 2	EPHA2	0.89	0.40	0.85	0.33	0.73	0.30	0.90	0.34	0.77	0.29	0.45	0.03	0.41	0.07
CD160 antigen	CD160	3.98	0.62	4.03	0.53	3.92	0.48	4.00	0.58	4.04	0.50	0.49	0.79	0.87	0.55
Tyrosine-protein kinase Lyn	LYN	1.58	0.82	1.64	0.83	1.69	0.71	1.57	0.78	1.60	0.75	0.49	0.49	0.98	0.87
Transforming growth factor alpha	TGF-alpha	0.88	0.62	0.91	0.52	0.87	0.55	0.73	0.62	0.62	0.46	0.51	0.94	0.04	0.00
Toll-like receptor 3	TLR3	4.14	0.79	4.06	0.83	4.14	0.70	4.13	0.76	4.12	0.76	0.53	0.78	0.81	0.69
Cornulin	CRNN	4.12	0.94	4.24	1.12	4.15	1.19	3.86	0.81	4.34	0.94	0.54	0.93	0.03	0.10
Endothelial cell-specific molecule 1	ESM-1	7.79	0.56	7.83	0.51	7.72	0.41	7.87	0.67	7.73	0.51	0.55	0.70	0.22	0.42
Tyrosine-protein kinase ABL1	ABL1	3.32	1.22	3.20	1.20	3.35	1.21	3.22	1.23	3.28	1.16	0.56	0.88	0.66	0.77
Melanoma-derived growth regulatory protein	MIA	9.14	0.36	9.13	0.32	9.09	0.32	9.20	0.35	9.18	0.30	0.57	0.33	0.13	0.38
C-type lectin domain family 4 member K	CD207	1.50	0.51	1.43	0.51	1.42	0.47	1.45	0.47	1.58	0.39	0.57	0.32	0.44	0.33
Receptor tyrosine-protein kinase erbB-2	ERBB2	5.66	0.48	5.71	0.40	5.59	0.32	5.69	0.37	5.68	0.42	0.60	0.21	0.69	0.71
Fc receptor-like B	FCRLB	0.76	0.65	0.78	0.62	0.67	0.58	0.81	0.72	0.58	0.44	0.61	0.71	0.67	0.12
Kallikrein-8	hK8	5.02	0.60	5.11	0.51	5.01	0.55	4.99	0.53	5.15	0.46	0.63	0.42	0.64	0.37
TGF-beta receptor type-2	TGFR-2	6.16	0.56	6.15	0.47	5.95	0.43	6.17	0.50	6.02	0.50	0.63	0.01	0.80	0.07
A disintegrin and metalloproteinase with thrombospondin motifs 15	ADAM-TS 15	2.92	0.53	2.89	0.56	2.76	0.49	3.00	0.49	3.00	0.49	0.70	0.19	0.16	0.35
Alpha-taxilin	TXLNA	4.60	1.41	4.50	1.41	4.66	1.43	4.54	1.51	4.50	1.29	0.73	0.75	0.96	0.55
Carboxypeptidase E	CPE	2.55	0.41	2.57	0.43	2.58	0.51	2.55	0.43	2.60	0.39	0.75	0.78	0.75	0.69
CD27 antigen	CD27	7.09	0.57	7.13	0.51	7.01	0.43	6.90	0.49	6.87	0.46	0.75	0.42	0.01	0.02
CD70 antigen	CD70	2.56	0.60	2.56	0.53	2.40	0.44	2.64	0.59	2.43	0.54	0.77	0.12	0.41	0.17
Furin	FURIN	2.83	0.41	2.85	0.47	2.63	0.42	2.75	0.46	2.61	0.64	0.77	0.02	0.20	0.00
Tumor necrosis factor receptor superfamily member 4	TNFRSF4	2.26	0.64	2.34	0.79	2.17	0.51	2.21	0.63	2.08	0.52	0.79	0.53	0.66	0.13
Granzyme B	GZMB	2.27	1.21	2.22	1.07	2.33	1.42	2.29	1.11	2.13	0.98	0.85	0.39	0.59	0.76
Folate receptor gamma	FR-gamma	7.04	2.33	7.03	2.31	6.38	1.89	6.68	1.86	6.92	2.35	0.86	0.07	0.79	0.10
Vimentin	VIM	2.80	1.11	2.70	0.99	2.62	1.25	2.49	0.99	2.27	1.16	0.86	0.42	0.06	0.00
T-cell leukemia / lymphoma protein 1A	TCL1A	4.69	1.74	4.53	1.75	4.89	2.03	4.86	1.37	4.76	1.38	0.87	0.60	0.19	0.46
C-X-C motif chemokine 13	CXCL13	7.94	0.76	8.05	0.99	7.84	0.92	7.73	0.78	7.67	0.82	0.89	0.31	0.04	0.02
FAS-associated death domain protein	FADD	1.50	1.06	1.50	1.04	1.58	1.04	1.31	1.08	1.51	1.05	0.91	0.70	0.24	0.93
Mucin-16	MUC-16	3.15	1.34	3.07	1.16	2.87	1.20	2.66	0.92	2.85	1.00	0.91	0.46	0.02	0.34
Xaa-Pro aminopeptidase 2	XPNPEP2	5.44	1.02	5.43	1.02	5.36	0.96	5.31	0.91	5.65	0.77	0.93	0.66	0.13	0.46
Cyclin-dependent kinase inhibitor 1	DKN1A	3.51	1.71	3.54	1.66	3.50	1.54	3.54	1.66	3.52	1.57	0.93	0.99	0.82	0.90
Annexin A1	ANXA1	1.76	1.02	1.65	0.75	1.75	1.03	1.42	0.63	1.56	0.98	0.94	0.68	0.02	0.07

NPX: normalized protein expression
LAC: lung adenocarcinoma
benign*: benign samples where inflammatory related diseases were excluded
SD: standard deviation
CRC met: colorectal metastasis
↑: upregulated in LAC

↓: downregulated in LAC
The grey cells indicate protein values with an adjusted p-val <0.05
