# Supplementary material for: Multiplex plasma protein profiling identifies novel markers to discriminate patients with adenocarcinoma of the lung
Source: BMC Cancer. 2019 Jul 29;19:741. doi: 10.1186/s12885-019-5943-3 (PMC6664554; doi:10.1186/s12885-019-5943-3)
Supplement: Supplementary file 2 — Pseudo code: Pseudo code for the TreeBagger algorithm, which was used to develop a multi-parameter classificator. (DOCX 14 kb) [file 12885_2019_5943_MOESM2_ESM.docx]

**Additional file 2**. Pseudocode

Used variables from Tree object created by Matlabs TreeBagger function (found in Tree.Trees{1,n})

e.g: [2,3] = Tree.Trees{1,2}.Children(1,:).

In the pseudo code allTreeData is the Tree object containing all trees. In this code we iterate through allTreeData extracting relevant parameters together with the 3 best desired predictors for each tree to Tree(n).

getStats(allTreeData)

{

For n = 1:length(allTreeData)

{

  #get the variables from allTreeData and assign them to stats

Tree(n).Stats

{

    Parent = allTreeData.Trees(n).Parent

    Children = allTreeData.Trees(n).Children

    NodeClass = allTreeData.Trees(n).NodeClass

    IsBranchNode = allTreeData.Trees(n).IsBranchNode

    ClassProbability = allTreeData.Trees(n).ClassProbability

    NodeSize = allTreeData.Trees(n).NodeSize

    ClassCount = allTreeData.Trees(n).ClassCount

    CutPredictor = allTreeData.Trees(n).CutPredictor

    CutPoint = allTreeData.Trees(n).CutPoint

}

#Get all nodes from the third level of tree nodes

Tree(n).Stats=Tree(n)Stats(Parent>3)

Tree(n).Stats=Tree(n)Stats(Parent<8)

#Find all trees having one node with classProbability <95% and exclude rest

Tree(n)Stats=Tree(n).Stats(classProbability > 95%)

#Find largest classCount for each tree for both classification groups

Tree(n).Stats=Tree(n).Stats(classCount == max(classCount))

Tree(n).Stats.cutPredictor

currentCutPoint = Tree(n).Stats.cutPoint

getParentPredictors(Tree(n).Stats.cutPredictor ,Tree(n).Stats.cutPoint,Tree(n))

{

#First predictor = Parent(1)

Tree(n).predictor(1) = Tree(n).Parent(1)

Tree(n).predictor(1).cutPoint=Parent(1).cutPoint

#Second Predictor is either Parent (2) or (3)

If (Tree(n).Parent(2).Children.cutPredictor==Stats.cutPredictor)

{

Tree(n).predictor(2) = Tree(n).Parent(2)

Tree(n).predictor(2).cutPoint=Parent(2).cutPoint

}

else

{

Tree(n).predictor(2) = Tree(n).Parent(3)

Tree(n).predictor(2).cutPoint=Parent(3).cutPoint

}

Tree(n).predictor(3) = Tree(n).Stats.cutPredictor

Tree(n).predictor(3).cutPoint=Tree(n).Stats.cutPoint

}

}

}
